# Crystal structure of bis­[4-(4-chloro­benz­yl)pyridine-κ*N*]bis­(thio­cyanato-κ*N*)zinc

**DOI:** 10.1107/S160053681402039X

**Published:** 2014-09-27

**Authors:** Julia Werner, Tristan Neumann, Inke Jess, Christian Näther

**Affiliations:** a Institut für Anorganische Chemie, Christian-Albrechts-Universität Kiel, Max-Eyth-Strasse 2, 24118 Kiel, Germany

**Keywords:** crystal structure, zinc complex, thio­cyanate, tetra­hedral coordination, hydrogen bonding

## Abstract

In the crystal structure of the title compound, [Zn(NCS)_2_(C_12_H_10_ClN)_2_], the Zn^2+^ cation is *N*-coordinated by two terminally bonded thio­cyante anions and by two 4-(4-chloro­benz­yl)pyridine ligands within a slightly distorted tetra­hedron. The asymmetric unit consists of half of the discrete complex, the central Zn^2+^ cation of which is located on a twofold rotation axis. The discrete complexes are linked into layers *via* a weak inter­molecular hydrogen-bonding inter­action, with a H⋯Cl distance of 2.85 Å and a C—H⋯Cl angle of 151°. These layers extend parallel to the *ab* plane and are held together by dispersion forces only.

## Related literature   

For related crystal structures with thio­cyanate ligands and Zn^2+^ in a tetra­hedral coordination sphere, see: Fettouhi *et al.* (2002 ▶); Kong *et al.* (2010 ▶); Zhu *et al.* (2008 ▶).
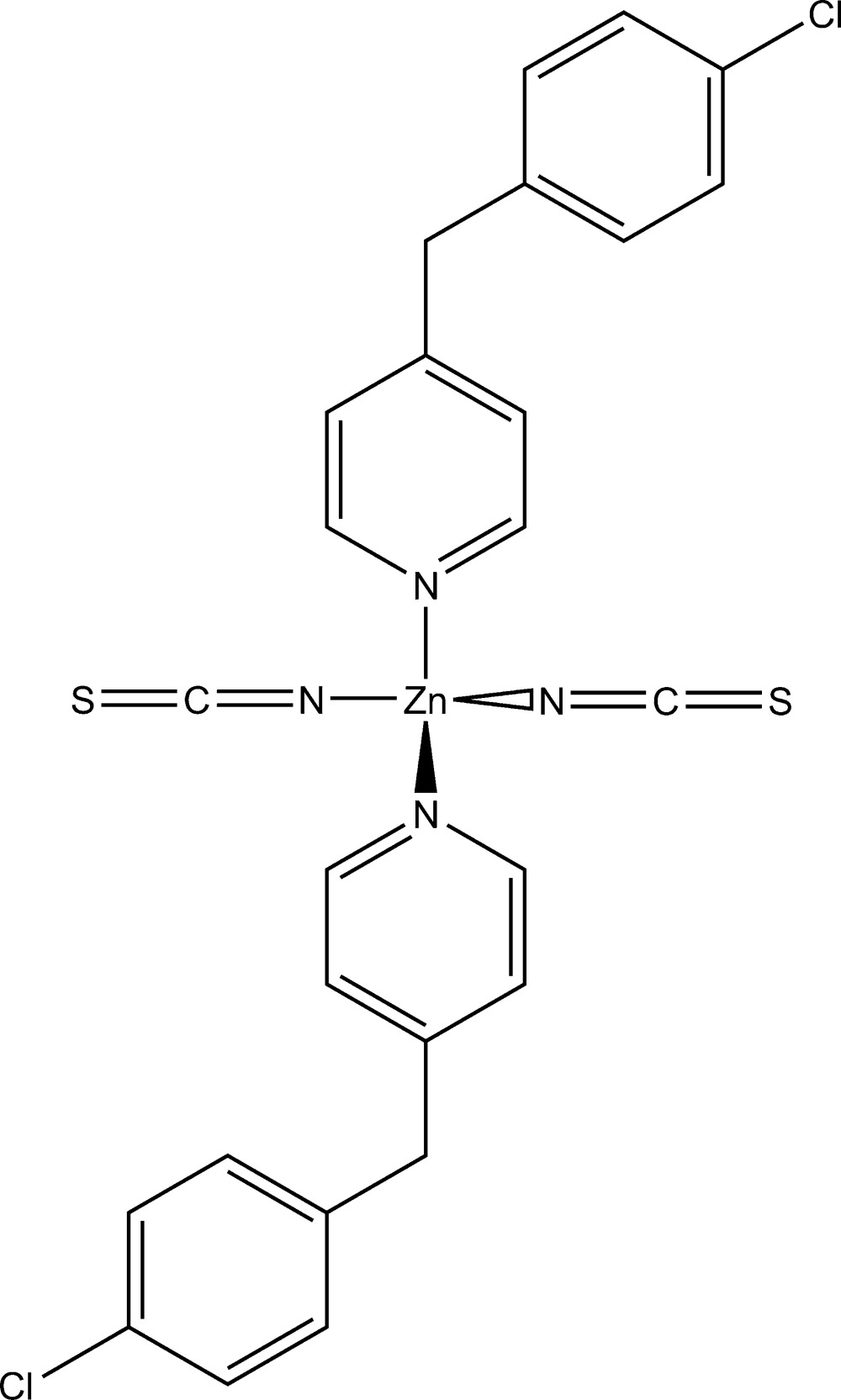


## Experimental   

### Crystal data  

[Zn(NCS)_2_(C_12_H_10_ClN)_2_]
*M* *_r_* = 588.85
Monoclinic, 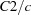

*a* = 29.094 (3) Å
*b* = 4.9911 (3) Å
*c* = 18.312 (2) Å
β = 98.867 (8)°
*V* = 2627.3 (4) Å^3^
*Z* = 4
Mo *K*α radiation
μ = 1.32 mm^−1^
*T* = 150 K
0.12 × 0.08 × 0.07 mm

### Data collection  

Stoe IPDS-2 diffractometer
Absorption correction: numerical (*X-SHAPE* and *X-RED32*; Stoe, 2008 ▶) *T*_min_ = 0.879, *T*_max_ = 0.906
8309 measured reflections
2570 independent reflections
1773 reflections with *I* > 2σ(*I*)
*R*_int_ = 0.077

### Refinement  

*R*[*F* ^2^ > 2σ(*F* ^2^)] = 0.063
*wR*(*F* ^2^) = 0.148
*S* = 1.08
2570 reflections
159 parameters
H-atom parameters constrained
Δρ_max_ = 0.68 e Å^−3^
Δρ_min_ = −0.46 e Å^−3^

### 

Data collection: *X-AREA* (Stoe, 2008 ▶); cell refinement: *X-AREA*; data reduction: *X-AREA*; program(s) used to solve structure: *SHELXS97* (Sheldrick, 2008 ▶); program(s) used to refine structure: *SHELXL97* (Sheldrick, 2008 ▶); molecular graphics: *XP* in *SHELXTL* (Sheldrick, 2008 ▶) and *DIAMOND* (Brandenburg, 2011 ▶); software used to prepare material for publication: *publCIF* (Westrip, 2010 ▶).

## Supplementary Material

Crystal structure: contains datablock(s) I, global. DOI: 10.1107/S160053681402039X/wm5054sup1.cif

Structure factors: contains datablock(s) I. DOI: 10.1107/S160053681402039X/wm5054Isup2.hkl

**Fig. 1.**
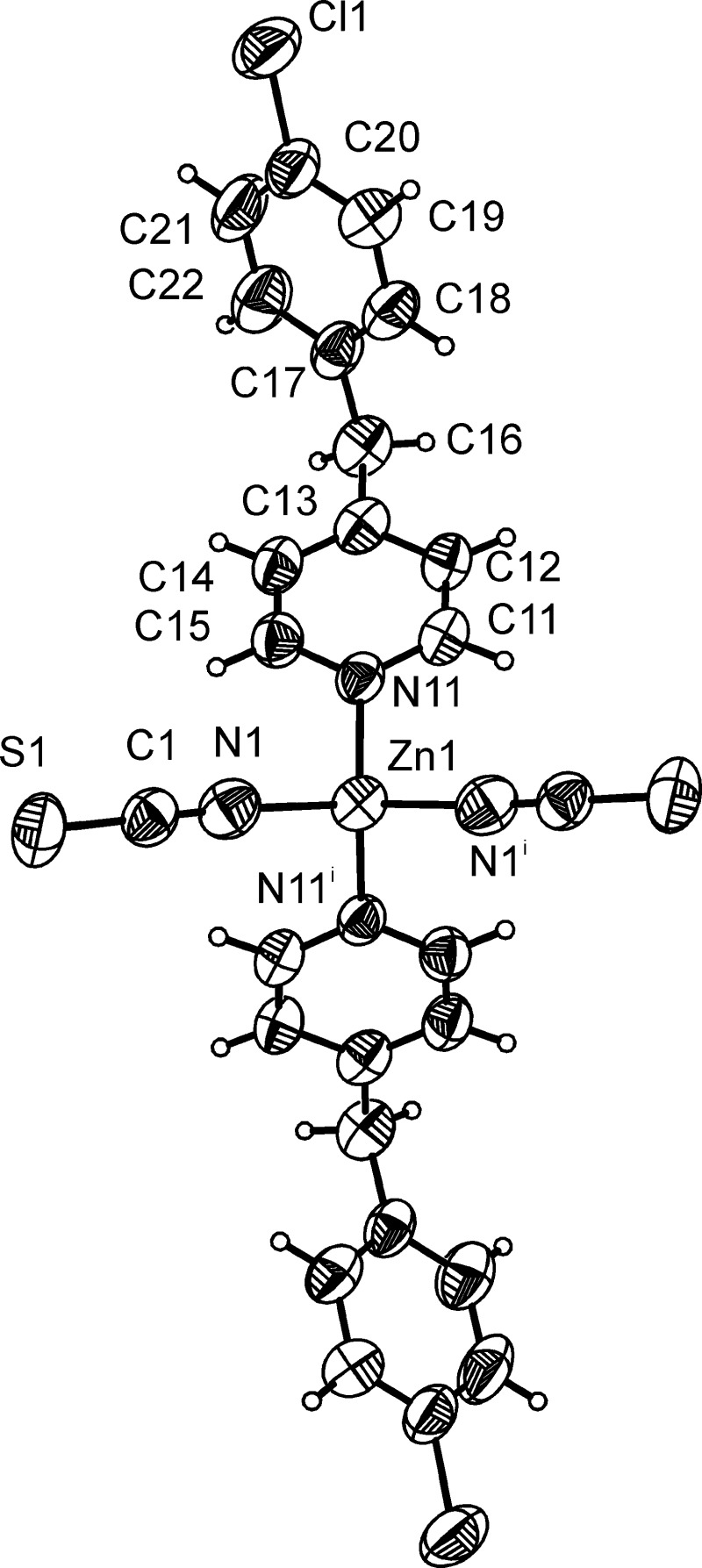
Molecular structure of the title compound with atom labelling and displacement ellipsoids drawn at the 50% probability level. [Symmetry code: i) -x, y, -z+1/2.]

**Fig. 2.**
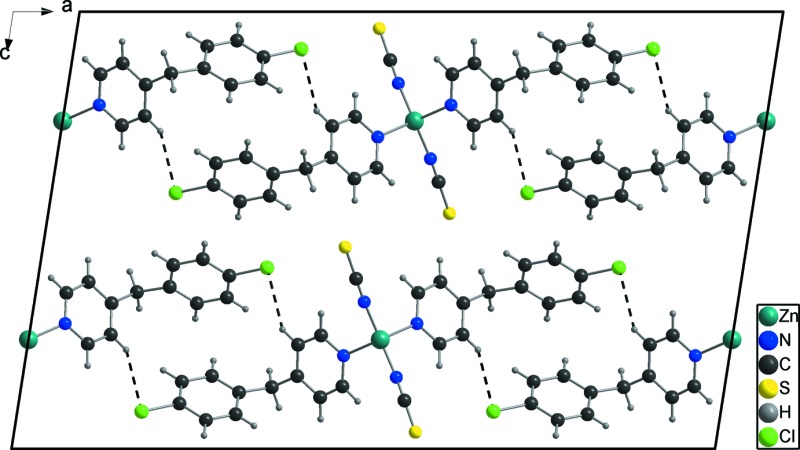
Crystal structure of the title compound in a projection along the b axis.

. DOI: 10.1107/S160053681402039X/wm5054fig1.tif
Mol­ecular structure of the title compound with atom labelling and displacement ellipsoids drawn at the 50% probability level. [Symmetry code: i) −x, y, −z+1/2.]

b . DOI: 10.1107/S160053681402039X/wm5054fig2.tif
Crystal structure of the title compound in a projection along the *b* axis.

CCDC reference: 1021253

Additional supporting information:  crystallographic information; 3D view; checkCIF report

## supplementary crystallographic information

### S1. Synthesis and crystallization

ZnSO_4_·H_2_O was purchased from Merck and 4-(4-chloro­benzyl)­pyridine and Ba(NCS)_2_·3H_2_O were purchased from Alfa Aesar. Zn(NCS)_2_ was synthesized by stirring 17.5 g (57.00 mmol) Ba(NCS)_2_·3H_2_O and 10.23 g (57.00 mmol) ZnSO_4_·H_2_O in 300 mL water at RT for three hours. The white residue of BaSO_4_ was filtered off, and the solution was evaporated by heating. The homogeneity of the product was investigated by X-ray powder diffraction and elemental analysis. The title compound was prepared by the reaction of (0.6 mmol) 108.9 mg Zn(NCS)_2_ and (0.15 mmol) 105.5 µL 4-(4-chloro­benzyl)­pyridine in 1.5 mL aceto­nitrile at RT. After few days, colorless needle-like crystals of the title compound were obtained.

### S2. Refinement

Hydrogen atoms were positioned with idealized geometry and were refined with *U*_iso_(H) = 1.2 *U*_eq_(C) using a riding model with C—H = 0.95 Å for aromatic and C—H = 0.99 Å for methyl­ene H atoms.

### Crystal data

**Table:**

[Zn(NCS)_2_(C_12_H_10_ClN)_2_]	*Z* = 4
*M**_r_* = 588.85	*F*(000) = 1200
Monoclinic, *C*2/*c*	*D*_x_ = 1.489 Mg m^−^^3^
Hall symbol: -C 2yc	Mo *K*α radiation, λ = 0.71073 Å
*a* = 29.094 (3) Å	θ = 1.4–26.0°
*b* = 4.9911 (3) Å	µ = 1.32 mm^−^^1^
*c* = 18.312 (2) Å	*T* = 150 K
β = 98.867 (8)°	Needle, colourless
*V* = 2627.3 (4) Å^3^	0.12 × 0.08 × 0.07 mm

### Data collection

**Table:**

Stoe IPDS-2 diffractometer	2570 independent reflections
Radiation source: fine-focus sealed tube	1773 reflections with *I* > 2σ(*I*)
Graphite monochromator	*R*_int_ = 0.077
ω scans	θ_max_ = 26.0°, θ_min_ = 1.4°
Absorption correction: numerical (*X-SHAPE* and *X-RED32*; Stoe, 2008)	*h* = −35→35
*T*_min_ = 0.879, *T*_max_ = 0.906	*k* = −6→6
8309 measured reflections	*l* = −22→21

### Refinement

**Table:**

Refinement on *F*^2^	Primary atom site location: structure-invariant direct methods
Least-squares matrix: full	Secondary atom site location: difference Fourier map
*R*[*F*^2^ > 2σ(*F*^2^)] = 0.063	Hydrogen site location: inferred from neighbouring sites
*wR*(*F*^2^) = 0.148	H-atom parameters constrained
*S* = 1.08	*w* = 1/[σ^2^(*F*_o_^2^) + (0.0577*P*)^2^ + 3.849*P*] where *P* = (*F*_o_^2^ + 2*F*_c_^2^)/3
2570 reflections	(Δ/σ)_max_ < 0.001
159 parameters	Δρ_max_ = 0.68 e Å^−^^3^
0 restraints	Δρ_min_ = −0.46 e Å^−^^3^

### Special details

**Table:**

Geometry. All esds (except the esd in the dihedral angle between two l.s. planes) are estimated using the full covariance matrix. The cell esds are taken into account individually in the estimation of esds in distances, angles and torsion angles; correlations between esds in cell parameters are only used when they are defined by crystal symmetry. An approximate (isotropic) treatment of cell esds is used for estimating esds involving l.s. planes.
Refinement. Refinement of F^2^ against ALL reflections. The weighted R-factor wR and goodness of fit S are based on F^2^, conventional R-factors R are based on F, with F set to zero for negative F^2^. The threshold expression of F^2^ > 2sigma(F^2^) is used only for calculating R-factors(gt) etc. and is not relevant to the choice of reflections for refinement. R-factors based on F^2^ are statistically about twice as large as those based on F, and R- factors based on ALL data will be even larger.

### Fractional atomic coordinates and isotropic or equivalent isotropic displacement parameters (Å^2^)

**Table:**

	*x*	*y*	*z*	*U*_iso_*/*U*_eq_	
Zn1	0.0000	−0.30960 (18)	0.2500	0.0536 (3)	
N1	0.03121 (14)	−0.4936 (9)	0.3362 (3)	0.0632 (11)	
C1	0.04787 (16)	−0.6043 (11)	0.3893 (3)	0.0564 (12)	
S1	0.07036 (5)	−0.7701 (3)	0.46152 (9)	0.0767 (5)	
N11	0.04855 (12)	−0.0763 (8)	0.2133 (2)	0.0482 (9)	
C11	0.04345 (16)	0.0091 (10)	0.1431 (3)	0.0547 (12)	
H11	0.0185	−0.0612	0.1088	0.066*	
C12	0.07260 (16)	0.1938 (10)	0.1181 (3)	0.0552 (11)	
H12	0.0671	0.2525	0.0682	0.066*	
C13	0.11000 (16)	0.2929 (10)	0.1667 (3)	0.0551 (12)	
C14	0.11577 (16)	0.2014 (12)	0.2384 (3)	0.0591 (13)	
H14	0.1411	0.2640	0.2731	0.071*	
C15	0.08515 (16)	0.0205 (10)	0.2597 (3)	0.0561 (12)	
H15	0.0899	−0.0395	0.3096	0.067*	
C16	0.14365 (18)	0.4920 (11)	0.1412 (3)	0.0654 (14)	
H16A	0.1505	0.6354	0.1786	0.078*	
H16B	0.1289	0.5759	0.0944	0.078*	
C17	0.18905 (16)	0.3592 (11)	0.1292 (3)	0.0600 (14)	
C18	0.18905 (17)	0.1565 (13)	0.0792 (3)	0.0688 (15)	
H18	0.1603	0.0952	0.0529	0.083*	
C19	0.22994 (19)	0.0380 (15)	0.0660 (4)	0.0815 (18)	
H19	0.2292	−0.1021	0.0307	0.098*	
C20	0.27132 (18)	0.1235 (16)	0.1039 (4)	0.0776 (18)	
C21	0.2727 (2)	0.3210 (18)	0.1536 (4)	0.093 (2)	
H21	0.3017	0.3783	0.1800	0.112*	
C22	0.2313 (2)	0.4443 (15)	0.1669 (4)	0.0854 (19)	
H22	0.2324	0.5859	0.2018	0.103*	
Cl1	0.32321 (5)	−0.0187 (5)	0.08551 (11)	0.1091 (8)	

### Atomic displacement parameters (Å^2^)

**Table:**

	*U*^11^	*U*^22^	*U*^33^	*U*^12^	*U*^13^	*U*^23^
Zn1	0.0483 (4)	0.0500 (5)	0.0615 (5)	0.000	0.0050 (3)	0.000
N1	0.058 (2)	0.057 (3)	0.074 (3)	0.006 (2)	0.008 (2)	0.004 (2)
C1	0.051 (2)	0.055 (3)	0.061 (3)	−0.003 (2)	0.003 (2)	−0.007 (3)
S1	0.0709 (8)	0.0855 (12)	0.0657 (9)	−0.0042 (7)	−0.0148 (7)	0.0094 (8)
N11	0.0429 (18)	0.050 (2)	0.050 (2)	0.0061 (17)	0.0016 (16)	−0.0037 (19)
C11	0.048 (2)	0.053 (3)	0.059 (3)	0.001 (2)	−0.004 (2)	−0.002 (2)
C12	0.053 (2)	0.051 (3)	0.058 (3)	−0.001 (2)	−0.004 (2)	0.004 (3)
C13	0.051 (2)	0.041 (3)	0.071 (3)	0.005 (2)	0.001 (2)	−0.006 (3)
C14	0.052 (2)	0.063 (3)	0.059 (3)	−0.002 (2)	−0.004 (2)	−0.007 (3)
C15	0.055 (3)	0.058 (3)	0.053 (3)	0.001 (2)	0.004 (2)	−0.005 (2)
C16	0.064 (3)	0.048 (3)	0.081 (4)	−0.007 (2)	0.003 (3)	−0.002 (3)
C17	0.050 (3)	0.061 (4)	0.066 (3)	−0.011 (2)	−0.003 (2)	0.014 (3)
C18	0.049 (3)	0.076 (4)	0.078 (4)	−0.008 (3)	−0.001 (2)	0.001 (3)
C19	0.061 (3)	0.103 (5)	0.080 (4)	0.009 (3)	0.008 (3)	0.000 (4)
C20	0.050 (3)	0.110 (5)	0.069 (4)	0.004 (3)	−0.001 (3)	0.027 (4)
C21	0.051 (3)	0.132 (6)	0.089 (5)	−0.024 (4)	−0.012 (3)	0.034 (5)
C22	0.066 (3)	0.091 (5)	0.093 (5)	−0.017 (3)	−0.006 (3)	−0.003 (4)
Cl1	0.0565 (8)	0.168 (2)	0.1023 (13)	0.0255 (10)	0.0118 (8)	0.0551 (14)

### Geometric parameters (Å, º)

**Table:**

Zn1—N1^i^	1.928 (5)	C15—H15	0.9500
Zn1—N1	1.928 (5)	C16—C17	1.524 (7)
Zn1—N11	2.024 (4)	C16—H16A	0.9900
Zn1—N11^i^	2.024 (4)	C16—H16B	0.9900
N1—C1	1.157 (6)	C17—C18	1.363 (8)
C1—S1	1.611 (6)	C17—C22	1.380 (7)
N11—C11	1.342 (6)	C18—C19	1.383 (8)
N11—C15	1.346 (6)	C18—H18	0.9500
C11—C12	1.377 (7)	C19—C20	1.363 (8)
C11—H11	0.9500	C19—H19	0.9500
C12—C13	1.386 (7)	C20—C21	1.338 (10)
C12—H12	0.9500	C20—Cl1	1.747 (6)
C13—C14	1.375 (7)	C21—C22	1.408 (10)
C13—C16	1.518 (7)	C21—H21	0.9500
C14—C15	1.367 (7)	C22—H22	0.9500
C14—H14	0.9500		
			
N1^i^—Zn1—N1	123.1 (3)	C14—C15—H15	118.6
N1^i^—Zn1—N11	105.49 (17)	C13—C16—C17	112.0 (4)
N1—Zn1—N11	106.33 (16)	C13—C16—H16A	109.2
N1^i^—Zn1—N11^i^	106.32 (16)	C17—C16—H16A	109.2
N1—Zn1—N11^i^	105.49 (17)	C13—C16—H16B	109.2
N11—Zn1—N11^i^	109.7 (2)	C17—C16—H16B	109.2
C1—N1—Zn1	176.6 (4)	H16A—C16—H16B	107.9
N1—C1—S1	177.6 (5)	C18—C17—C22	118.2 (5)
C11—N11—C15	116.9 (4)	C18—C17—C16	120.6 (4)
C11—N11—Zn1	121.4 (3)	C22—C17—C16	121.2 (6)
C15—N11—Zn1	121.4 (3)	C17—C18—C19	121.6 (5)
N11—C11—C12	123.2 (4)	C17—C18—H18	119.2
N11—C11—H11	118.4	C19—C18—H18	119.2
C12—C11—H11	118.4	C20—C19—C18	119.5 (7)
C11—C12—C13	119.3 (5)	C20—C19—H19	120.2
C11—C12—H12	120.4	C18—C19—H19	120.2
C13—C12—H12	120.4	C21—C20—C19	120.6 (6)
C14—C13—C12	117.5 (5)	C21—C20—Cl1	119.6 (5)
C14—C13—C16	121.5 (5)	C19—C20—Cl1	119.7 (6)
C12—C13—C16	121.0 (5)	C20—C21—C22	120.2 (6)
C15—C14—C13	120.3 (5)	C20—C21—H21	119.9
C15—C14—H14	119.9	C22—C21—H21	119.9
C13—C14—H14	119.9	C17—C22—C21	119.9 (7)
N11—C15—C14	122.9 (5)	C17—C22—H22	120.1
N11—C15—H15	118.6	C21—C22—H22	120.1

Symmetry code: (i) −*x*, *y*, −*z*+1/2.
