# Supplementary figures and images for: Crystal structure of bis­[4-(4-chloro­benz­yl)pyridine-κN]bis­(thio­cyanato-κN)zinc
Source: Acta Crystallogr Sect E Struct Rep Online. 2014 Sep 27;70(Pt 10):m355–6. doi: 10.1107/S160053681402039X (PMC4257228; doi:10.1107/S160053681402039X)

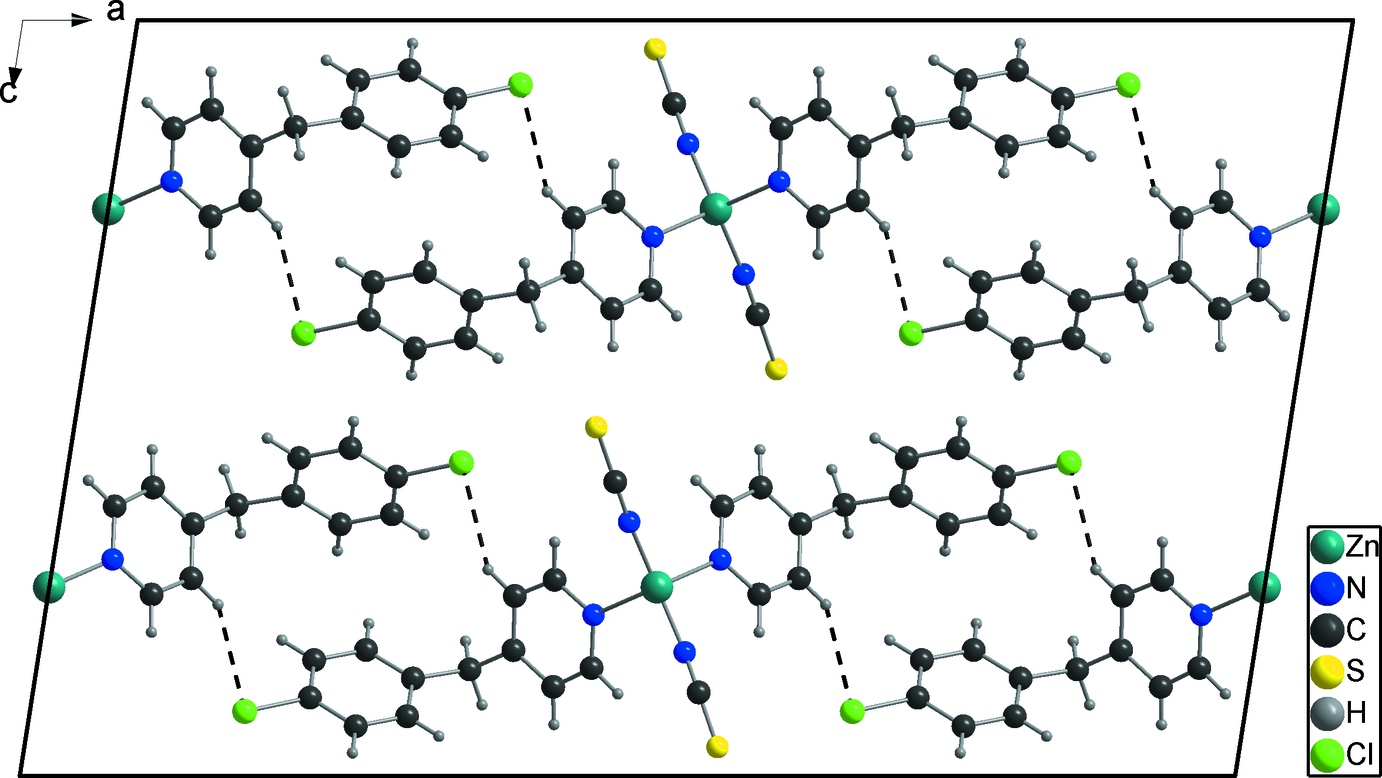

Supplement: Supplementary file 4 [file e-70-0m355-fig2.tif]
